# Patient and clinician's ratings of improvement in methadone-maintained patients: Differing perspectives?

**DOI:** 10.1186/1477-7517-8-23

**Published:** 2011-08-26

**Authors:** Joan Trujols, Núria Siñol, Ioseba Iraurgi, Francisca Batlle, Joan Guàrdia, José Pérez de los Cobos

**Affiliations:** 1 Unitat de Conductes Addictives, Servei de Psiquiatria, Hospital de la Santa Creu i Sant Pau, Institut d'Investigació Biomèdica Sant Pau (IIB Sant Pau), Barcelona, Spain; 2 DeustoSalud - R&D&Innovation in Clinical and Health Psychology, University of Deusto, Bilbao, Spain; 3 Departament de Metodologia de les Ciències del Comportament, Facultat de Psicologia, Universitat de Barcelona, Barcelona, Spain

## Abstract

**Background:**

In the last few years there seems to be an emerging interest for including the patients' perspective in assessing methadone maintenance treatment (MMT), with treatment satisfaction surveys being the most commonly-used method of incorporating this point of view. The present study considers the perspective of patients on MMT when assessing the outcomes of this treatment, acknowledging the validity of this approach as an indicator. The primary aim of this study is to evaluate the concordance between improvement assessment performed by two members of the clinical staff (a psychiatrist and a nurse) and assessment carried out by MMT patients themselves.

**Method:**

Patients (n = 110) and their respective psychiatrist (n = 5) and nurse (n = 1) completed a scale for assessing how the patient's condition had changed from the beginning of MMT, using the Patient Global Impression of Improvement scale (PGI-I) and the Clinical Global Impression of Improvement scale (CGI-I), respectively.

**Results:**

The global improvement assessed by patients showed weak concordance with the assessments made by nurses (Quadratic-weighted kappa = 0.13, p > 0.05) and by psychiatrists (Quadratic-weighted kappa = 0.19, p = 0.0086), although in the latter, concordance was statistically significant. The percentage of improved patients was significantly higher in the case of the assessments made by patients, compared with those made by nurses (90.9% vs. 80%, Z-statistic = 2.10, p = 0.0354) and by psychiatrists (90.9% vs. 50%, Z-statistic = 6.48, p < 0.0001).

**Conclusions:**

MMT patients' perception of improvement shows low concordance with the clinical staff's perspective. Assessment of MMT effectiveness should also focus on patient's evaluation of the outcomes or changes achieved, thus including indicators based on the patient's experiences, provided that MMT aim is to be more patient centred and to cover different needs of patients themselves.

## Background

Methadone maintenance treatment (MMT) should be considered as a specific psychopharmacological treatment of heroin dependence [1] at the same time as being an essential and fundamental element of harm reduction strategies [2]. Since its introduction in heroin dependence management, MMT has undergone a constant process of review and evaluation. During the past fifteen years there have been a substantial number of systematic reviews aiming at methodically and rigorously summarising available scientific evidence on the efficacy of this treatment (e.g., [1-4]). In all these systematic reviews, efficacy and effectiveness of MMT have been evaluated almost exclusively using so-called hard indicators or criteria [5,6]: retention on the programme, abstinence from non-prescription opioids, reduced morbidity and mortality and/or reduced crime rate, among other variables. However, assessment of MMT should include patient's subjective evaluation of the treatment process and the changes achieved, by means of indicators based on their experiences [7].

At a more global level, several authors have pointed out the importance of including patient's perspective when assessing health technologies and health services [8,9], and the potential significance of patient's perspective towards research and generation of knowledge [10]. It should also be noted that there is a growing interest in incorporating patient's perspective in the development of clinical practice guidelines [11,12]. In fact, some of the most recent systems for establishing the strength of the recommendations (e.g., the GRADE approach [13]) include patient's preferences as one of the determinant factors: the higher the variability (i.e. uncertainty) of patients' values and preferences, the higher the probability of a weak recommendation. However, the majority of clinical practice guidelines do not include evidence on patient's preferences, particularly given the limited number of studies available on this issue [14]. Notable exceptions are the Allergic Rhinitis and Its Impact on Asthma guidelines (ARIA) [15] which include in their recommendations, suggestions such as "in many patients with strong preference for the oral versus the intranasal route of administration, an alternative choice may be reasonable" [15], p. 471.

MMT patients' perspective has received scant attention from clinicians and researchers [16,17]. Traditionally, patients' point of view has not been considered a relevant contribution to the design, implementation and evaluation of MMT. However, over the last few years there seems to be an emerging interest for including patients' perspectives in assessing MMT. This new framework is probably related to the gradual, albeit timid tendency to modify the hierarchised doctor-patient relationship based on the traditional medical model of disease, which implies a biased and exclusive perception of drug users as non-competent persons [18,19].

To date, MMT satisfaction surveys have been the most commonly-used method of incorporating patient's perspective [7]. However, other attempts have considered and explored variables such as beliefs and views about methadone [20], perceived adjustment of the methadone dose [21], assessment of the relational dynamic established with other stakeholders [22], degree of participation in decision making [21], indicators of perceived quality [23] and views on the development and improvement of MMT [24]. Assessment of MMT outcomes from patient's perspective (i.e., evaluation of global perceived improvement in comparison with the pre-MMT situation) has not received the attention it deserves. To our knowledge, only one study [25] has recently and explicitly addressed this issue but without taking into account clinician's point of view about the same outcome, nor the concordance between both perspectives.

This study considers the perspective of patients on MMT when assessing the outcomes of this treatment, acknowledging its validity as a relevant indicator. The primary aim of this study is to evaluate the concordance between improvement assessment performed by two members of the clinical staff (psychiatrists and nurses) and assessment carried out by MMT patients themselves. It also analyses whether perceived improvement by MMT patients is associated with their satisfaction with treatment and with their views on methadone as a medication for treating heroin dependence.

## Method

### Participants

Units of analysis in this study were 110 cases whose information was provided by three sources: 110 patients on MMT and their respective nurses and psychiatrists. Participants were methadone-maintained, opioid-dependent patients who had received MMT at our centre for at least 3 months, and who had signed an informed consent form. Each MMT patient has a psychiatrist who is responsible for their assessment, diagnosis, prescription and treatment, and a nurse in charge of monitoring and assessing patient status, handling daily medical issues and dispensing methadone. A total of five psychiatrists and one nurse participated in the study, with an average of 22 patients (range 2-46) per psychiatrist.

### Instruments

To assess how the patient's condition had changed from the beginning of MMT, patients and their respective psychiatrist and nurse completed a scale: the Patient Global Impression of Improvement scale (PGI-I) and the Clinical Global Impression of Improvement scale (CGI-I) [26], respectively. These scales assess the degree in which the patient has improved in comparison with the pre-MMT situation, and consist of a single item in a 7-point Likert format (1 = very much improved to 7 = very much worse). An additional rating analysis developed by Demyttenaere et al. [27] was also used: improved (points 1 and 2), stable (points 3 to 5), and worsened (points 6 and 7). Satisfaction with MMT was assessed with the Verona Service Satisfaction Scale for Methadone Treatment (VSSS-MT) [28]. This scale has 27 items and consists of four factors: basic interventions, specific interventions, social worker skills, and psychologist skills. Items are based on a 5-point Likert scale (1 = terrible, 2 = mostly dissatisfied, 3 = mixed, 4 = mostly satisfied, 5 = excellent). The ranges of clinical significance for the VSSS-MT scores are [29]: 1-2 (very dissatisfied), > 2-3 (slightly dissatisfied), > 3-4 (slightly satisfied), and > 4-5 (very satisfied). Patients' opinion of methadone as a medication to treat heroin dependence was explored with the following question [21]: 'Taking into account your overall experience, what is your impression about methadone as a medication for carrying out maintenance treatment of heroin dependence?'. The same 5-point Likert scale used as a response format in the VSSS-MT (1 = terrible to 5 = excellent) is established for this question.

### Procedure

The research project was approved by the Clinical Research Ethics Committee of the Hospital de la Santa Creu i Sant Pau (Barcelona, Spain). A research psychologist invited eligible patients to participate in the study and administered the surveys, which were conducted without the presence of clinical staff. No compensation was offered for participating in the study. When the clinicians completed the CGI-I scale for each patient, they were blind to patient's score on the PGI-I. The surveys were conducted between January and March 2007.

### Data analysis

Quadratic-weighted kappa coefficients (κ_w_) were used to express the concordance between the clinical staff's and patient's own assessment of improvement. Z tests for comparing two proportions were used to determine whether the percentage of improved patients was statistically different when comparing the assessments made by clinicians with those made by the patients themselves. Kendall's tau-b coefficients (τ*b*) were calculated to examine the association between improvement assessed by the patient and the VSSS-MT scores or the patient's opinion of methadone as a medication. All statistical tests were two-tailed and considered significant if p < 0.05. Results for the quadratic-weighted Kappa coefficients and Kendall tau-b coefficients were assessed using the ranges for effect size interpretation recommended by Ferguson [30]. Statistical analyses were performed using Epidat 3.1 (Dirección Xeral de Saúde Pública de la Conselleria de Sanidade de la Xunta de Galicia and Pan American Health Organization) and SPSS Statistics 17.0 (SPSS Inc., Chicago, IL).

## Results

### Acceptance and completion of the survey

Although there were 120 patients on MMT at our centre at the time the study was performed, four had been on treatment for less than three months and so they were not invited to take part. Of the 116 surveys proposed, 110 (94.8%) patients agreed to participate and answered the survey. However, a psychiatrist did not complete the corresponding CGI-I scale for two patients. There were no statistically significant differences regarding the sociodemographic and clinical characteristics of the patients who refused to participate in the survey, compared with those who accepted.

### Characteristics of the participants and MMT

The 110 patients who completed the survey ranged in age from 22 to 58 years, with a mean of 39.4 years (standard deviation [SD] = 6.5). Males accounted for 73.6% of the sample. With regard to marital status, 63.3% were single, 19.3% married or living with a partner, 13.8% separated or divorced and 3.7% widowed. Participants used or had used heroin intravenously (56.9%), by inhalation (18.3%) or by snorting (24.8%). The mean of patients' total MMT episodes was 1.6 (SD = 0.9, range = 1-6). Participants were taking a methadone dose of (mean ± SD) 67.2 ± 39.8 mg/d (range = 5-180). A percentage of 43.5 of patients were taking < 60 mg/d of methadone, 42.4% were taking 60-100 mg/d, and 14.1% were taking > 100 mg/d.

### Relationship between patient and clinical staff assessment of improvement

Concordance between global improvement assessed by psychiatrists and nurse was moderate and statistically significant (κ_w _= 0.40, 95% Confidence Interval [CI] = 0.26-0.55, p < 0.0001). However, the global improvement assessed by patients showed low concordance with the assessments made by nurse (κ_w _= 0.13, 95% CI = -0.05-0.31, p > 0.05; Table 1) and psychiatrists (κ_w _= 0.19, 95% CI = 0.08-0.30, p = 0.0086; Table 2), although the latter value reached statistical significance.

**Table 1:** Concordance between patient and nurse perceptions of improvement

		Patient Global Impression of Improvement (PGI-I) scale							
		Very much improved	Much improved	Minimally improved	No change	Minimally worse	Much worse	Very much worse	Total
	**Very much improved**	61	12	1	1			2	77
	**Much improved**	8	2		1				11
	**Minimally improved**	4	2	1		1	1		9
**Clinical Global Impression of Improvement (CGI-I) scale**	**No change**	4	4	1	1				10
	**Minimally worse**	1							1
	**Much worse**	1							1
	**Very much worse**	1							1
	**Total**	80	20	3	3	1	1	2	110

Data expressed as frequencies.

**Table 2:** Concordance between patient and psychiatrist perceptions of improvement

		Patient Global Impression of Improvement (PGI-I) scale							
		Very much improved	Much improved	Minimally improved	No change	Minimally worse	Much worse	Very much worse	Total
	**Very much improved**	29	4						33
	**Much improved**	15	5	1					21
	**Minimally improved**	21	4			1	1	2	29
**Clinical Global Impression of Improvement (CGI-I) scale**	**No change**	8	4	2	2				16
	**Minimally worse**	4	1						5
	**Much worse**	1	2						3
	**Very much worse**				1				1
	**Total**	78	20	3	3	1	1	2	108

Data expressed as frequencies.

By recoding scores of PGI-I and CGI-I into three categories (improved, stable and worsened) [27], the percentage of improved patients was significantly higher in the case of the patients' own assessments compared with those made by nurse (90.9% vs. 80%, 95% CI for the difference between proportions = 0.01-0.21, Z-statistic = 2.10, p = 0.0354) and psychiatrists (90.9% vs. 50%, 95% CI for the difference between proportions = 0.29-0.53, Z-statistic = 6.48, p < 0.0001).

### Relationship between patient-assessed improvement and their satisfaction with MMT

The global score in the VSSS-MT was (mean ± SD) 3.8 ± 0.5, and VSSS-MT factor scores were as follows: 3.9 ± 0.6 in basic interventions, 3.5 ± 0.7 in specific interventions, 3.5 ± 1.0 in social worker skills, and 3.9 ± 0.9 in psychologist skills. All these scores indicated 'slight satisfaction' according to the VSSS-MT ranges of significance [29]. No statistically significant association was found between perceived improvement by MMT patients and their satisfaction with treatment. With regard to overall VSSS-MT score and factors, the Kendall tau-b values (-0.12 < τ*b *< 0.09) did not reach statistical significance (Table 3).

**Table 3:** Kendall's tau-b correlations of PGI-I with VSSS-MT scores and opinion of methadone as a medication

	PGI-I
**VSSS-MT, overall**	-0.032
**VSSS-MT, Basic interventions**	-0.001
**VSSS-MT, Specific interventions**	-0.063
**VSSS-MT, Social worker skills**	-0.106
**VSSS-MT, Psychologist skills**	0.088
**Opinion of methadone as a medication**	-0.178*

VSSS-MT: Verona Service Satisfaction Scale for Methadone Treatment; PGI-I: Patient Global Impression of Improvement scale; *p < 0.05, two-tailed. The scores on the PGI-I scale are in descending order of improvement (1 = very much improved to 7 = very much worse) while both the VSSS-MT and the scores on the opinion on methadone as a medication are in increasing order of satisfaction (1 = terrible to 5 = excellent).

### Relationship between patient-assessed improvement and patient's opinion of methadone as a medication

Although most participants had an excellent (27.3%) or mostly satisfied (56.4%) opinion of methadone as a medication for treating opioid dependence, almost a sixth of the participants expressed an opinion that was mixed (10.9%), mostly dissatisfied (1.8%) or terrible (3.6%). A statistically significant but weak association was found between perceived improvement by patients on MMT and their opinions of methadone as a medication (τ*b *= -0.18, p = 0.042, Table 3). In fact, this correlation lost its statistical significance when patient-perceived improvement was classified by just three categories (improved, stable and worsened) [27]: τ*b *= -0.13, p > 0.05.

## Discussion

The primary objective of this study was to assess the concordance between the assessment of improvement evaluated by two members of clinical staff (a psychiatrist and a nurse) and the assessment performed by MMT patients themselves. The results reveal that global improvement assessed by patient showed low concordance with nurse- and psychiatrist-assessments. It has to be mentioned that although patient-psychiatrist concordance reached statistical significance, it did not meet the recommended minimum effect size [30]. This low concordance between clinical staff's and patient's perspective stems from the fact that patients' assessments are significantly more frequently positive. This patient-clinician discrepancy regarding perceived improvement is consistent with the results of the study conducted by Pulford et al. [31] comparing client (new admissions to an alcohol and other drug counseling community-based service) and clinician perspectives on problem improvement at two months of follow-up. Similarly, the main results of this study showed that clinician ratings of client improvement were significantly lower than ratings the clients gave themselves [31]. This patient-clinician discrepancy regarding patient improvement is also quite common, although not unanimous, in the field of mental health (see [32] for a review).

A first possible explanation for this patient-clinician discrepancy regarding the assessment of MMT results could lie in the fact that patients may have a tendency to assess their progress more positively. This tendency would in turn be explained by one of the following facts or possibilities, which are not mutually exclusive: a) patients have a more detailed knowledge of their situation before starting MMT and of their current condition and changes in relation to the previous situation, b) some clinicians may be too strict or demanding when allocating certain scores, c) some patients aware of their limited or lack of improvement, could overrate the improvement in order to reduce their cognitive dissonance.

An alternative explanation for the patient-clinician discrepancy regarding the assessment of MMT results could stem from the fact that clinicians and patients emphasise different areas and outcomes when they define success, progress or improvement. This definition would largely depend on how MMT is conceptualised and what goals have been set for this treatment. As Koester et al. [33] showed, there are many different goals or reasons for a heroin user to enter and/or stay on MMT and they may differ from those of the professionals who provide these treatments. In turn, these professionals also have a wide range of beliefs and attitudes about the goal of MMT [34]. However, all too often the goal of MMT is not negotiated and agreed with patient [35,36], implicitly and naively assuming that both the goal and, consequently, the definition of improvement are the same for the patient and clinician. Despite this assumption, a considerable number of authors (e.g., [7,33,37-39]) point out the need to consider patients' priorities when a) rethinking what is understood by a successful MMT or intervention, and b) establishing areas and outcomes that need to be assessed when establishing the effectiveness of these interventions. In fact, within some harm reduction services or programs, models and instruments to assess outcomes have been developed that are consistent with the fact that both the concepts of success and progress and their assessment should be reformulated in order to reflect the patient's perspective (e.g., [40,41]).

Another notable finding of the present study is that MMT patients' perceived improvement is not associated with their satisfaction with MMT or with their views on methadone as a medication. These findings are consistent with previous results on a sample of patients on MMT by Perreault et al. [25] and by Ries et al. [42] in a study based on a sample of dually diagnosed outpatients participating in a long-term integrated dual disorder treatment. Correlational analyses of Perreault et al. [25] between several measures of perceived improvement and satisfaction with MMT revealed both non-significant associations and some statistically significant but weak correlations. All in all, these results seem to show that satisfaction with MMT is weakly related to the treatment outcome when assessed from patient's perspective. Therefore, it could be suggested that these two variables, though slightly related, represent distinct approaches to MMT patient's perspective. In this regard, a study by Rademakers et al [43] found that, when exploring the extent to which satisfaction aspects determine patients' overall satisfaction rating, aspects related to process were the most important predictors of this global rating, followed by aspects of structure and, lastly, aspects of outcome.

This study is not without limitations. Its cross-sectional design does not permit causal relationships to be established between the different variables related to the patient's perspective. Although the sampling was exhaustive, the fact that patients and clinicians were from a single MMT centre, limits the generalisability of these findings to other MMT programs. Since both the PGI-I scale and the CGI-I scale are generic single-item tools, the detail of information obtained from these scales is somewhat limited. Moreover, due to the fact that both scales do not specify criteria for assessing the change experienced by patients with regard to their condition before starting MMT, it cannot be assured whether patients and clinicians have been guided by the same outcome areas and goals when assessing this change. However in the case of the PGI-I scale, this very fact may have increased the relevance, significance or content validity of a scale developed by clinicians without the participation of patients themselves. Despite these limitations, the fact that parallel versions of the same scale (i.e., PGI-I and CGI-I) were used in this study to assess MMT patients' improvement, reinforces the robustness of the low concordance between patients and clinical staff when rating perceived improvement of MMT patients, found in this study.

Further research is needed not only to assess the generalisability of these findings to patients and clinicians from other MMT programs and/or geographic areas, but also to provide new data about patient-clinician concordance regarding MMT outcomes by including additional instruments for a deeper assessment of the matter. Moreover, future studies should also provide evidences, through qualitative research methods such as focal groups, in-depth interviews or cognitive interviews, about the outcome domains and/or variables that both patients and clinicians consider in assessing patient's improvement.

## Conclusions

MMT patients' perception of improvement shows low concordance with the clinical staff's perspective. Assessment of MMT efficacy and effectiveness should also take into account patient's evaluation of the outcomes or changes achieved. Therefore, indicators based on the patient's experiences should be included, provided that MMT aim is to be more patient centred and to cover different needs of patients themselves.

## Abbreviations

CGI-I: Clinical Global Impression of Improvement scale; 95% CI: 95% Confidence Interval; MMT: methadone maintenance treatment; PGI-I: Patient Global Impression of Improvement scale; SD: standard deviation; VSSS-MT: Verona Service Satisfaction Scale for Methadone Treatment.

## Competing interests

The authors declare that they have no competing interests.

## Authors' contributions

JT and JP designed the study and wrote the protocol. JT and NS managed the literature searches and summaries of previous related work. Data collection was done by FB and NS. JT, II and JG designed the analysis plan. JT and II undertook the statistical analysis. All authors participated in the interpretation of findings. JT wrote the first draft of the manuscript. All authors contributed to and have approved the final version submitted for publication.
